# Dissemination and Implementation Research Funded by the US National Institutes of Health, 2005–2012

**DOI:** 10.1155/2013/909606

**Published:** 2013-03-27

**Authors:** Mindy Tinkle, Richard Kimball, Emily A. Haozous, George Shuster, Robin Meize-Grochowski

**Affiliations:** UNM College of Nursing, 1 University of New Mexico MSC 095350, Albuquerque, NM 87131-0001, USA

## Abstract

Dissemination and implementation (D&I) research is a growing area of science focused on overcoming the science-practice gap by targeting the distribution of information and adoption of interventions to public health and clinical practice settings. This study examined D&I research projects funded under specific program announcements by the US National Institutes of Health (NIH) from 2005 to 2012. The authors described the projects' D&I strategies, funding by NIH Institute, focus, characteristics of the principal investigators (PIs) and their organizations, and other aspects of study design and setting. Results showed 46 R01s, 6 R03s, and 24 R21s funded totaling $79.2 million. The top funders were the National Cancer Institute and the National Institute of Mental Health, together providing 61% of funding. The majority of PIs were affiliated with Schools of Medicine or large, nonprofit research organizations and think tanks. Only 4% of projects were to PIs with appointments at Schools of Nursing, with 7% of the funding. The most commonly funded projects across all of the studies focused on cancer control and screening, substance abuse prevention and treatment, and mental health services. Typically implemented in community and organizational settings, D&I research provides an excellent opportunity for team science, including nurse scientists and interdisciplinary collaborators.

## 1. Introduction

The existence of a gap between science and practice is universally recognized. Clinical research findings and clinical practice guidelines that have promise to improve health move very slowly from the research setting into clinical practice, and many of these interventions never reach those who could benefit. It is estimated that it takes an average of 17 years to translate 14% of original research into benefit for patients and an average of 9 years for interventions recommended as evidence-based practices to be fully adopted [1, 2].

Dissemination and implementation (D&I) research is a growing area of science focused on overcoming this science-practice gap. Research on dissemination addresses the targeted distribution or spread of information and interventions to specific public health and clinical practice settings. Implementation science is the study of methods to promote the integration of evidence and change practice patterns and health care policy within real-world public health and clinical service settings [3]. Using the language from models depicting the continuum of translational science from bench to practice and health impact, D&I research is often depicted as “T3” and “T4” science [4, 5].

Although the research enterprise has generated a rich supply of evidence-based interventions, programs, and services, knowledge about how to best disseminate and implement these evidence-based practices has not kept pace. Evidence is needed for how these interventions can be “scaled up,” what contextual factors and conditions are pivotal to successful adoption, and how to give added attention to issues of external validity, fidelity, and sustainability. D&I nurse researchers and practitioners are important players in advancing the goals of this science to improve patient and system outcomes [6–8].

 Evidence does suggest that passive approaches to dissemination, such as the publication of consensus statements in professional journals, mass mailings, and untargeted presentations to heterogeneous groups, are ineffective strategies to achieve significant uptake and practice change [9, 10]. Targeted and active dissemination strategies, such as hands-on technical assistance, replication guides, point-of-decision prompts, and training workshops with hands-on experience, are more promising [11]. Implementation strategies have been described as either “top down” or “bottom up” and include a range of approaches, such as stakeholder relationship building and communication, continuous quality management, audit and feedback, service delivery practices and training, and local consensus building [12]. These D&I strategies are often directed at multiple levels and in different combinations of levels, including patient, provider, organizational, and policy. Features of organizations (e.g., hospitals, clinics, workplaces, and schools), such as organizational leadership and climate, managerial relations, and absorptive capacity, are increasingly seen as key intervention targets to facilitate D&I efforts [13].

Although D&I research in health is a relatively young science, advances in both the rigor and ambitiousness of studies over the past decade reflect robust growth in the field [14]. Many conceptual frameworks for guiding D&I research, such as the Reach, Effectiveness, Adoption, Implementation, and Maintenance (RE-AIM) model [15], have been developed and are being tested. Common themes in these frameworks include a heavy emphasis on context, fidelity adaptation and quality of implementation, multilevel targets, and engagement of the target population in partnership research [16]. Research is also focused on developing and validating sound measures important for D&I research, such as measures for key organizational-level constructs [13]. In addition, comparative effectiveness studies of competing active D&I strategies are beginning to appear in the literature, including evaluation of cost [17]. Study designs best suited to answer questions in D&I research are also developing, including a focus on mixed-methods designs and system science approaches [18].

 Federal funding for D&I research has traditionally been very small, particularly in relation to the funding available for discovery research. Although the portfolio of D&I research at the National Institutes of Health (NIH) is growing, funding for this science remains extremely small compared with the $30 billion each year that the NIH spends on basic and efficacy research [19]. In 2005, a trans-NIH committee (including the National Institute of Nursing Research [NINR]) issued the first of a set of multi-institute program announcements to stimulate research in this area. These program announcements have been continuously reissued over the past 8 years and include the Research Project Grant (R01) and Small Grant (R03 and R21) mechanisms. The purpose of this funding opportunity is to support innovative approaches to identifying, understanding, and overcoming barriers to the adaptation, adoption, and integration of evidence-based interventions and guidelines that previous research has shown to be efficacious and effective, but where uptake to date has been limited or significantly delayed [20].

A total of 76 D&I research projects have been funded by the NIH through these multi-institute program announcements from 2005 to 2012. Research findings from some of these NIH-funded projects have been presented at the NIH Annual Conference on the Science of Dissemination and Implementation and published in the literature. However, no summary of these funded projects examining the body of work in this research portfolio is available.

 The purpose of this study was to examine the D&I research projects funded through these program announcements from 2005 to 2012 in terms of describing what has been funded by which NIH Institutes, the dollars invested, characteristics of the principal investigators (PIs) and their organizations, and an assessment of the focus of these projects, the D&I strategies employed, and other aspects of study design and setting. Although the studies funded through these Program Announcements Reviewed (PARs) do not include all the possible D&I-related research projects funded by NIH over this period, this study was confined to these projects because they represent outcomes of a sustained, multi-institute initiative to stimulate development of D&I science. This paper presents a description of these funded projects and suggests opportunities for nurse scientists in D&I research.

## 2. Methods

**Figure 1:**
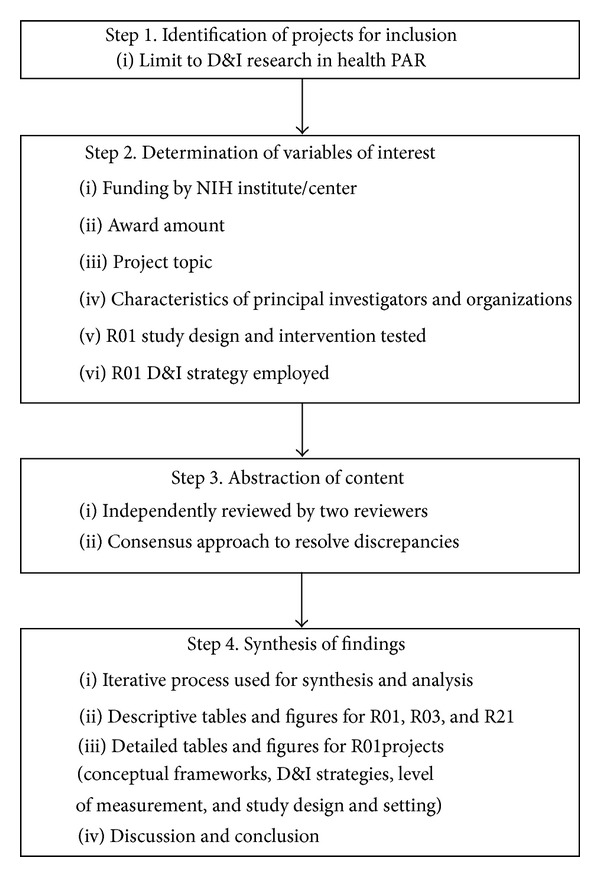
Flowchart for the review process. PAR: Program Announcement Reviewed; D&I: Dissemination and Implementation.

 To accomplish the aims of this study, the abstracts from all projects funded by the NIH under the multi-institute “Dissemination and Implementation Research in Health” (PAR-06-039, PAR-06-520, PAR-06-521, PAR-07-086, PAR-10-038, PAR-10-039, and PAR-10-040) were accessed through the NIH Research Portfolio Online Reporting Tools (RePORT) [21]. Projects funded under an earlier program announcement specific to mental health (PAR-02-131, “Dissemination and Implementation Research in Mental Health”) were excluded from this review. The paper process is outlined in Figure 1.

The narrative abstracts for all 76 projects were independently examined by two reviewers to extract information on the following areas: funding institute, award amount, project topic, and characteristics of the PI and awardee organization. The 46 R01 projects were further independently examined by 3 reviewers for conceptual frameworks, D&I strategies, level of measurement, and study design and setting. In cases of discrepancies in recorded findings, an iterative process of abstract review was used until 100% agreement among the reviewers was reached. The small grant mechanisms (i.e., R03s and R21s) were excluded from the analysis of the R01s in this examination because the smaller projects were mainly needs assessments, small pilots, and instrument development studies and were generally not intervention research.

 Summary tables and figures were constructed, using type of funding mechanism (R01, R03, R21) for initial categorization of abstracts. The quantitative results (e.g., frequencies and means) and qualitative results (e.g., project topic and D&I strategy) provided a foundation for drawing conclusions about the funded D&I research and discussing the implications for nursing research. 

## 3. Results

### 3.1. Overview of the Projects

**Figure 2:**
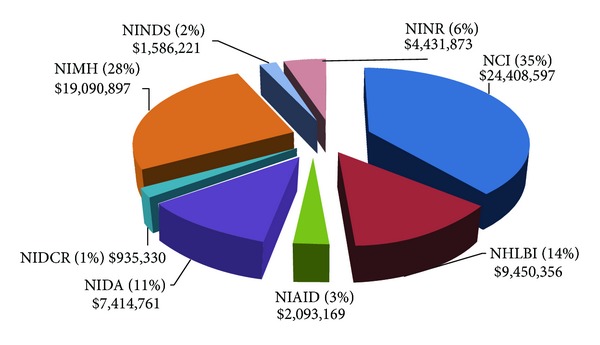
R01 funding by NIH Institutes, 2005–2012.

A total of 76 project abstracts were reviewed, representing 46 R01s, six R03s, and 24 R21s funded by the NIH, totaling $79.2 million during the 2005 to 2012 period (Table 1). Nine NIH Institutes and Centers funded these awards, with the National Cancer Institute (NCI) and the National Institute of Mental Health (NIMH) funding 58% of the total number of projects, which accounted for 61% of the total funding. The NINR awarded 6% of the projects: three R01s, one R03, and one R21, totaling $4.9 million. The NIH Fogarty International Center joined the program announcements in 2009 and funded several grants focused on global health.  Figure 2 depicts the funding for the R01 awards, again, with the majority of the larger research project grants funded by the NCI and the NIMH.

**Figure 3:**
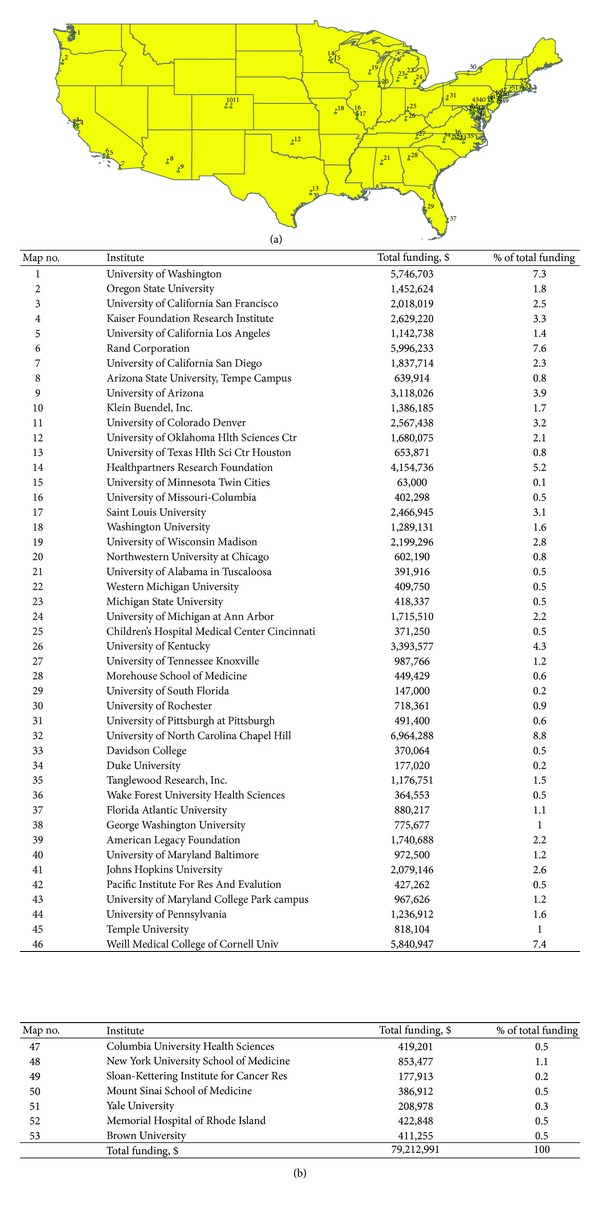
(a) Geographical distribution of institutions receiving project funding, (b) Legend for Figure 3(a).

The majority of PIs for these funded projects were affiliated with Schools of Medicine and large, nonprofit research organizations and think tanks, such as Rand and Kaiser, and these institutions received 58% of the total funding (Table 2). Schools of Public Health accounted for about 18% and 19% of the PI affiliations and the total funding, respectively. Only about 4% of the funded projects were to PIs at Schools of Nursing, making up about 7% of the total funding. The universities and research organizations funded for these projects were more heavily clustered in the West and East coastal states and the East North Central states (Figure 3). The institutions in the West and East coastal states received about two thirds of the total funding for all of the projects.

**Figure 4:**
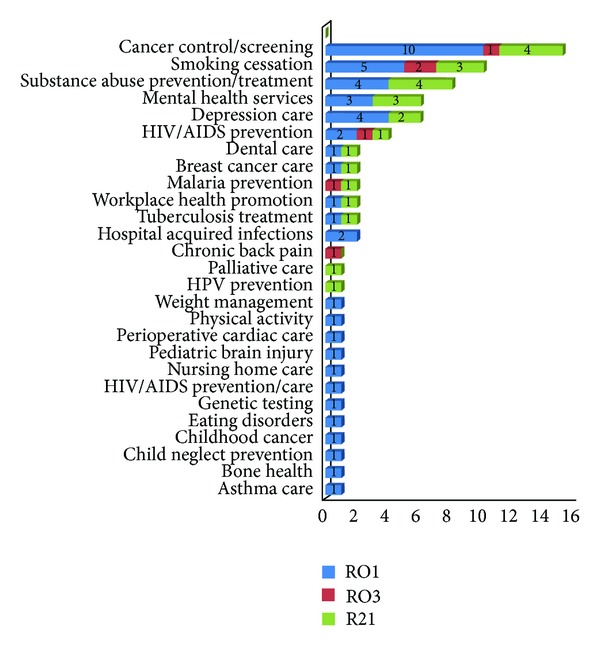
NIH R01, R03, and R21 study topics. HIV/AIDS: human immunodeficiency virus/acquired immunodeficiency syndrome; HPV: human papilloma virus.

The topic focus for the 76 projects funded over the past 8 years reflects a broad spectrum of areas, consistent with the missions of the sponsoring NIH Institutes and Centers. The projects included dissemination and implementation of evidence-based health behavior interventions and research-based guidelines, programs, and services for prevention, diagnosis, and clinical management in public health, clinical settings, and the public policy arena. As shown in Figure 4, the most commonly funded projects were cancer control and screening, substance abuse prevention and treatment, and mental health services.

Many of the projects involved scaling up interventions found to be effective in smaller trials or adapting an evidence-based intervention for implementation in a new setting. For example, one R01 project [22] studied the dissemination and implementation of an evidence-based weight-management program for veterans called MOVE! by scaling up this program to reach a broad population of veterans across the Veterans Affairs national network of medical centers and community clinics. Another R01 project [23] examined the impact of adapting and delivering an evidence-based organizational implementation strategy called Availability, Responsiveness, and Continuity (ARC), originally developed in Tennessee, on improving mental health services for youth in community-based agencies in a Midwest community.

### 3.2. A Focus on the R01 Projects

A more in-depth analysis of the R01 projects was conducted to better understand the intervention projects that move the science beyond the pilot phase. This analysis included an examination of the theories and frameworks, D&I strategies, levels of measurement, and study design and setting. 

#### 3.2.1. Theories and Frameworks

Among the 46 R01 projects, the range of orientation to a theory or model varied widely (Table 3). The majority of the R01-funded projects had no mention of a theoretical framework or guiding model (*n* = 22). Of the frameworks mentioned, the RE-AIM framework for evaluating interventions was most commonly utilized (*n* = 7), closely followed by Rogers' Diffusion of Innovations model (*n* = 5) [24]. One study combined Rogers' Diffusion of Innovations model and the RE-AIM framework (*n* = 1). Nine studies utilized frameworks that had specific relevance for their project, such as grants that proposed the use of behavioral interventions and had a related behavioral model supporting the intervention. For example, a project titled “Dissemination of a Theory-Based Bone Health Program in Online Communities” [25] utilized social cognitive theory in designing an online bone-health intervention targeting adults aged 50 years and older.

#### 3.2.2. D&I Strategies

Of the 46 R01 projects, 29 had one D&I strategy and 15 had two D&I strategies, for a total of 59 D&I strategies used (Table 4). In two of the abstracts, the strategies were either unclear or were not stated. Examining the D&I strategies, a majority (78%) of studies utilized active dissemination approaches, whereas 10% used passive dissemination approaches, and the remaining 12% used an evaluative approach (i.e., evaluation of an existing program). Many studies utilized a combination of mixed active, passive, and/or evaluative strategies. The range of active approaches varied; for example, one study adapted patient navigation strategies to Chinese women in Chicago [26] in an intervention that modified tailored patient navigation to improve cancer screening rates in this low-income and underserved population. The dissemination approach proposed in this study incorporated patient navigators as providers of cancer control education and screening in active teaching roles within the Asian immigrant population and was categorized in our study as hands-on technical assistance and training, two active D&I strategies. An example of evaluation of dissemination was demonstrated in a study examining smoking cessation and knowledge integration with people who use tobacco control quitlines [27], in which participant social network analysis was conducted to provide insight about potential dissemination approaches in the future.

#### 3.2.3. Levels of Measurement

**Figure 5:**
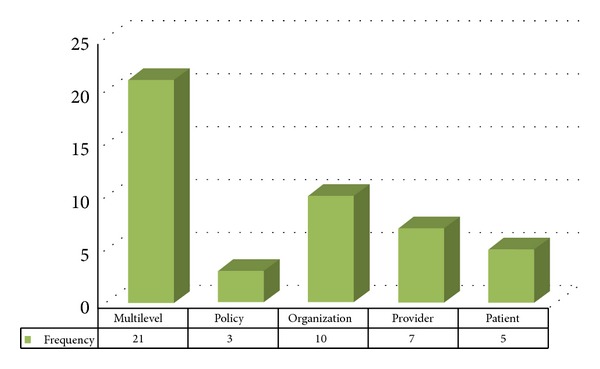
Frequencies for the level of measurement for the evaluation of intervention study outcomes.

These R01 projects used five levels of measurement in evaluating the intervention outcomes: policy level, patient level, provider level, organizational level, and multilevel outcome measures. Multilevel outcome measures involved different combinations of the other four levels, such as a combination of organizational and provider measures or an intervention study that measured both provider and patient outcomes. Multilevel outcome measures were the most common means of evaluation for these R01 projects (47%), whereas the most frequent individual outcome measure was at the organizational level (22%; see Figure 5).

One example of a research study using a multilevel outcomes methodology [28] involved a colorectal screening intervention replicated by a community health services agency for Asian American patients. Intervention outcomes were measured using a combination multilevel evaluation at both the organizational (i.e., the agency) and individual levels by collecting data from individual providers at the intervention community agencies.

An example of program evaluation using a single level of outcome measure [29] involved a 5-year project that examined the transportation, implementation, and sustainability of a computer-assisted cognitive-behavioral treatment (CACBT) for young children's anxiety in elementary schools. In this multisite study, each school provided at least four counselors, social workers, school psychologists, or teachers who could implement the intervention. The outcome of the project was measured in terms of whether or not the school personnel increased the use of the CACBT program and increased identification of elementary school students with distressing anxiety through use of the Behavioral Assessment System for Children, Second Edition, Teacher Rating Scale.

#### 3.2.4. Study Design and Setting

Many of these 46 R01 projects had multiple aims and multiple stages as part of the study intervention. The project designs included randomized controlled trials, quasiexperimental designs, case studies, survey research, community-based participatory approaches, and system science designs, such as social network analysis. 

Many projects utilized mixed-methods designs, using quantitative methods to measure outcomes and qualitative methods to describe processes or expand the depth of understanding. For example, one project [30] involved three separate stages, with the first stage using two in-depth case studies of model substance abuse treatment programs serving the substance abuser group in the community. The second stage used a quantitative telephone survey approach to collect data from the directors of all 480 substance abuse treatment programs serving substance abusers in their respective communities. The final stage involved a qualitative approach using in-depth case studies of 12 of these 480 substance abuse treatment programs from the stage-two telephone interviews. As a result, the researchers planned to collect, analyze, and report both quantitative and qualitative data gathered during the three stages of the project [30]. 

When these R01 projects were grouped by predominant design, 43% of the studies used a quasiexperimental design, and 24% used randomized controlled trials. Together, these two designs accounted for two out of three designs among the R01 studies (Table 5). The project settings were also diverse and included rural and urban primary care and specialty care practices (*n* = 14), state government and international health settings (*n* = 8), community health agencies (*n* = 7), hospitals (*n* = 4), online social networking (*n* = 4), schools (*n* = 4), large health care systems (*n* = 2), churches (*n* = 2), and worksites (*n* = 1). See Table 6 for a list of the 76 abstracts used in the study.

## 4. Discussion

 This review of abstracts was conducted to describe the R01, R03, and R21 projects funded under the NIH program announcements for D&I research from 2005 to 2012. Further analysis was performed for the R01 studies, which were intended to move the science beyond the pilot phase. 

 Review of these abstracts demonstrated a robust set of projects that reflect a growing and evolving area of science. NCI and NIMH were the major funders for the projects, which is indicative of their long history of working to advance this field. Each of these institutes has designated organizational D&I units and program officers dedicated to broadly stimulating this science. The PIs for these projects represented an array of disciplines, and the topical focus of the projects was equally diverse, illustrating the interdisciplinary nature of the D&I research community. Only a small proportion of PIs for these projects were from Schools of Nursing. Likewise, as illustrated in Figure 3, rural and western states were underrepresented among the funded projects.

When a theory or framework was present, it referred to one that is commonly applied in D&I research in the context of health, particularly the RE-AIM [15] and Diffusion of Innovations [24] models. Other commonly used models included those that supported individually focused behavioral interventions, such as cognitive behavioral theory. Consistent with the literature that demonstrates that active D&I strategies are more effective [12], most of the projects relied on active approaches, such as training and technical assistance. 

Assessment of the outcomes and fidelity of the dissemination and implementation of new preventive practices, guidelines, or programs in these projects were most often measured at multiple levels, such as the individual patient, the provider, and the organization. This multimodal approach is characteristic of more recent D&I research and may reflect an evolving use of systems thinking for D&I in terms of understanding how actors and organizations influence each other within a whole [31, 32]. The predominant study designs used in these projects included quasiexperimental and randomized clinical trials. Many of the projects used mixed methods, with a third of them including both quantitative and qualitative components. Mixed methods are particularly useful in generating data from multiple levels and many stakeholders and may be particularly suited to answer the complex questions in D&I research [18, 33].

 Many of these funded projects can be considered first-generation D&I research. What areas of knowledge development suggested from these studies and other experts should be promoted to move the field forward in terms of the next generation of D&I research? Developing a standard and consistent terminology for D&I research is one critical area requiring careful attention [33]. Additional theory development and testing are needed to better understand the relationships among the complex array of factors required for successful dissemination and implementation of health interventions in various settings [34]. Building a more robust set of common measures for D&I research is also a priority [13]. Glasgow and colleagues suggest that alternative study designs beyond the traditional randomized trial that emphasize the importance of external validity and that take advantage of existing social, environmental, and community data should be utilized [19]. A focus on D&I approaches with high-risk populations, including low-income, minority, and low-health literacy groups, and in low-resourced settings is also an imperative [19, 34]. Finally, new interdisciplinary collaborations with diverse partners, including key stakeholders, consumers, and clinicians, will be important to grow the science [19].

### 4.1. Implications for Nursing Research

D&I science moves beyond the individual as the unit of analysis to focus on groups, systems, the community, and beyond. Nursing research is conducted in all these areas, but D&I research is a way for nurses to influence health and health care on a larger scale. One example is the dissemination of evidence-based practice guidelines throughout a unit, hospital, and health care system. What are the best ways to have these guidelines widely used by nurses and other health care professionals? Which strategies are most cost-effective? 

 The D&I program announcements clearly present a potential funding opportunity for nurse scientists committed to translating evidence-based interventions to improve health (these program announcements were recently reissued on January 9, 2013, under PAR-13-055 (R01), PAR-13-054 (R21), and PAR-13-056 (R03)). Nursing has a rich history of work in research utilization to improve clinical care and promote practice-based inquiry. Nurse scientists are well prepared to lead and participate as members in interdisciplinary teams focused on disseminating and implementing evidence to practice. Symptom management and self-care in chronic disease and end-of-life care are just a few examples where nursing has made significant contributions to the science and should take a leadership role in translating this work to practice [35]. Many nurse researchers are skilled in both quantitative and qualitative designs often used in D&I research. A mixed-methods approach might be especially useful in testing different strategies for implementation across different populations. 

There are opportunities to learn more about D&I science and receive assistance and feedback on a proposed grant application. The NIH hosts an annual conference on the science of dissemination and implementation in health, where attendees can hear state-of-the science presentations, learn about research findings in the poster session, network with D&I scientists, and attend a technical assistance workshop led by NIH Program Officers and funded by D&I researchers. Other research training opportunities, such as the annual NIH-sponsored Training Institute on Dissemination and Implementation Research in Health, are also offered periodically to the extramural community. The NIH Office of Behavioral and Social Sciences Research website includes information about these opportunities (http://obssr.od.nih.gov/scientific_areas/translation/dissemination_and_implementation/index.aspx). 

Talking to the appropriate NIH Institute Program Officer identified on the PAR as the scientific contact person is also essential in planning an application submission. Considering the fit of an application with the mission of other institutes (e.g., NCI or NIMH), in addition to NINR, is also important. Grant applications for these PARs prior to 2010 were reviewed in Special Emphasis Panels, in which peer reviewers were appointed for each panel on a temporary basis. In 2010, the NIH Center for Scientific Review established a chartered study section to peer review these and other investigator-initiated applications in this science area, called the Dissemination and Implementation Research in Health Study Section (http://public.csr.nih.gov/StudySections/IntegratedReviewGroups/HDMIRG/DIRH/Pages/default.aspx). This website also provides a link to the study section's membership roster. Whereas standing members of this study section are appointed for a specific term, other temporary reviewers are often appointed for a specific review cycle to augment the scientific expertise that may be needed, depending on the pool of applications. Another invaluable opportunity for nurse scientists who have expertise in a specific area of D&I science is to volunteer to serve as a peer reviewer for this study section by sending their curriculum vitae and letter of interest to the Scientific Review Officer assigned to this study section. 

### 4.2. Limitations

The description and analysis in this review were based solely on the funded project abstracts published in the NIH RePORTER under the previously identified program announcements. By their nature, proposal abstracts present a limited amount of information and might not accurately represent the project once completed. Outcomes cannot be identified through review of proposal abstracts; this would require subsequent review of publications based on the funded projects. The level of analysis was limited by having access only to those abstracts publicly available through funding. Consequently, no conclusions can be made based on funded projects compared with all proposals submitted in response to this PAR. No information is publically available regarding the total number of applications submitted. Furthermore, it is not known how representative the funded projects are for any of the descriptors provided in this paper compared with submitted proposals. It is also not known whether different types of applications or specific topics were more or less likely to be funded in relation to the entire pool of applications.

 As reported by others [11, 12], this abstract review was hindered by inconsistent terminology for design and strategy and even for what was meant by dissemination or implementation. Although some abstracts provided details of the proposed projects, others omitted relevant content (e.g., model used). Equally evident from this review was the lack of common measures with established validity and reliability, particularly for measuring D&I processes and outcomes. The imperative to develop these common measures as key to the successful advancement of D&I science has been widely advocated [13, 34]. 

 Finally, it should be noted that D&I science has undergone much development since the first general program announcement was released in 2005. It is not known what impact this may have had on the number and quality of proposals submitted, especially during the latter part of the time selected for this review. 

## 5. Conclusion

 The overall goal of D&I science is to overcome the research-practice gap so that evidence-based health practices yield significant health benefits to all populations and across all health care settings [19]. The purpose of this paper was to enhance understanding of the body of work represented in the projects funded under the NIH dissemination and implementation program announcements over the past 8 years and suggest implications for nurse researchers. The projects in this portfolio demonstrated that D&I research is complex, often multiphase, and requires a collaborative, interdisciplinary approach. These projects make a highly significant contribution to the field, yet much work remains to be done, such as improving methods and measures, to move D&I science forward.

Although many nurse researchers and practitioners are engaged in D&I science, nurse scientists were underrepresented among the PIs for these projects. Nurse scientists are uniquely prepared to contribute to the advancement of D&I research in health. This NIH initiative represents an outstanding potential funding and leadership opportunity for nurse researchers committed to translational research and shortening the science-practice gap.

## Figures and Tables

**Table 1:** Dissemination and implementation awards by NIH Institute and grant mechanism, 2005–2012 (out-year funding for projects recently awarded is not included).

Institute	Mechanism	Funds, $ (%)	No. of awards (%)	Average award, $	% of total funding
	R01	24,408,597 (91)	19	1,284,663	
NCI	R03	213,000 (1)	2	106,500	
	R21	2,096,157 (8)	6	349,360	

	Totals	26,717,754 (100)	27 (36)		34

NIMH	R01	19,090,897 (89)	11	1,735,536	
	R21	2,353,747 (11)	6	392,291	

	Totals	21,444,644 (100)	17 (22)		27

	R01	7,414,761 (77)	4	1,853,690	
NIDA	R03	177,913 (2)	1	177,913	
	R21	2,041,179 (21)	5	408,236	

	Totals	9,633,853 (100)	10 (13)		12

NHLBI	R01	9,450,356 (100)	5 (7)	1,890,071	12

NIAID	R01	2,093,169 (67)	2	1,046,585	
	R21	1,035,998 (33)	4	259,000	

	Totals	3,129,167 (100)	6 (8)		4

	R01	4,431,873 (89)	3	1,477,291	
NINR	R03	166,404 (3)	1	166,404	
	R21	391,916 (8)	1	391,916	

	Totals	4,990,193 (100)	5 (7)		6

NIDCR	R01	935,330 (46)	1	935,330	
	R21	1,103,698 (54)	2	551,849	

	Totals	2,039,028 (100)	3 (4)		3

NINDS	R01	1,586,221 (100)	1 (1)	1,586,221	2

FIC	R03	221,775 (100)	2 (3)	110,888	0.3

Total funding					

	R01	69,411,204 (88)	46	1,508,939	
	R03	779,092 (1)	6	129,849	
	R21	9,022,695 (11)	24	375,946	

	Total	79,212,991 (100)	76		100

NCI: National Cancer Institute; NIMH: National Institute of Mental Health; NIDA: National Institute on Drug Abuse; NHLBI: National Heart, Lung, and Blood Institute; NIAID: National Institute of Allergy and Infectious Diseases; NINR: National Institute of Nursing Research; NIDCR: National Institute of Dental and Craniofacial Research; NINDS: National Institute of Neurological Disorders and Stroke; FIC: Fogarty International Center.

**Table 2:** Projects by PI affiliation, mechanism, and total funding.

PI affiliation	R01	R03	R21	No. of projects (%)	Funds, $ (%)
School of Medicine	21	3	8	32 (42)	29,066,546 (37)
Other research organizations (i.e., Rand, Kaiser, etc.)	7	1	4	12 (16)	17,518,075 (22)
School of Public Health	10	0	4	14 (18)	14,846,637 (19)
School of Nursing	3	0	0	3 (4)	5,869,637 (7)
School of Social Work	2	0	0	2 (3)	4,915,825 (6)
College of Arts and Sciences	2	1	3	6 (8)	3,308,268 (4)
Other university-based organizations	0	1	3	4 (5)	1,385,087 (2)
College of Biomedical Engineering	1	0	0	1 (1)	1,199,218 (2)
School of Dentistry	0	0	2	2 (3)	1,103,698 (1)

Totals	46	6	24	76 (100)	79,212,991 (100)

**Table 3:** Theories or models utilized in the R01 abstracts.

Rank (fewest to most)	Theory or model	Frequency (%)
1	Rogers' Diffusion of Innovations + RE-AIM	1 (2)
2	Nonspecific reference to theory or model	2 (4)
3	Rogers' Diffusion of Innovations (alone or in combination with another theory or model)	5 (11)
4	RE-AIM (alone or in combination with another theory or model)	7 (15)
5	Specific theoretical framework or model:	9 (20)
	Cooperation Extension System	1
	Community Readiness Model	1
	Quality Assurance Model	2
	Self-Regulation Theory of Health Behavior	1
	Collaborative Depression Core Model	1
	Cognitive Behavioral Theory	1
	Advanced Recovery Theory	1
	Program Change Model	1
6	No theory or model	22 (48)

Re-Aim: Reach, Effectiveness, Adoption, Implementation, and Maintenance.

**Table 4:** Passive, active, and evaluative D&I strategies identified.

Strategy/subcategory	No. (%)
Passive	
Publication of information, such as practice guidelines	6 (10)
Active	
Training (train the trainer, certificate training, and staff development workshop)	23 (39)
Hands-on technical assistance	8 (14)
Websites and interpersonal channels (social networking)	5 (8)
Replication guides	3 (5)
Phone calls	4 (7)
Social marketing	2 (3)
Point-of-decision prompts for use	1 (2)
Evaluative	
Evaluation	7 (12)

Total D&I strategies	59 (100)

**Table 5:** Predominant study designs and methods in the R01 projects.

Rank (most to fewest)	Predominant study design: 46 RO1 projects	Frequency (%)
1	Quasiexperimental designs	20 (43)
2	Randomized controlled trials	11 (24)
3	Systems science (e.g., social network analysis)	10 (22)
4	Case studies	3 (7)
5	Community-based participatory research	1 (2)
6	Unclear study design	1 (2)

Other categories		

	Studies with more than one design	14 (30)
	Studies using mixed methods (quantitative and qualitative)	14 (30)

**Table 6:** Abstracts evaluated in the study.

Principal investigator	Project title	Project number	Mechanism	Funding institute	Funding amount*
Aarons, Gregory	Leadership development for evidence-based practice implementation	1R21MH082731-01A1	R21	NIMH	$467,057
Aarons, Gregory et al.	Interagency collaborative teams to scale up evidence-based practice	1R01MH092950-01A1	R01	NIMH	$1,370,657
Allen, Rebecca Sue	Legacy intervention family enactment (LIFE): an effectiveness trial	1R21NR011112-01	R21	NINR	$391,916
Auerbach, Andrew D.	Improving use of perioperative beta-blockers with a multidimensional QI program	1R01HL086473-01	R01	NHLBI	$2,018,019
Berenholtz, Sean M.	A multifaceted intervention to reduce ventilator-associated pneumonia in the ICU	1R01HL105903-01A1	R01	NHLBI	$972,500
Bickell, Nina A.	Implementing cancer treatment measuring and reporting in office and hospital practice	1R21CA132773-01A2	R21	NCI	$419,201
Bogner, Hillary R.	Implementing care for depression and diabetes	1R21MH094940-01A1	R21	NIMH	$240,000
Botvin, Gilbert J.	A collaborative system approach for the diffusion of evidence-based prevention	1R01DA023437-01A1	R01	NIDA	$3,640,886
Bradbury, Angela R.	Communicating genetic test results by telephone: a randomized trial	1R01CA160847-01A1	R01	NCI	$668,104
Brownson, Ross C.	Cancer control dissemination research among state-level policy makers	1R01CA124404-01A1	R01	NCI	$2,466,945
Brownson, Ross C.	Disseminating evidence-based interventions to control cancer	1R01CA160327-01A1	R01	NCI	$517,465
Bruce, Martha L.	Homecare agency-randomized trial of web implementation strategy for depression	1R01MH096441-01A1	R01	NIMH	$386,912
Campbell, Marci K.	Dissemination of a weight-management program among US veterans	1R01CA124400-01	R01	NCI	$2,813,567
Cates, Joan Roberts	Optimizing HPV vaccination: parents, providers, and preteen boys	1R21AI095590-01A1	R21	NIAID	$407,000
Clarke, Jennifer Grace et al.	Methods for understanding sentinel events	1R21DA032739-01	R21	NIDA	$422,848
Cobb, Nathan	Online social networks for dissemination of smoking cessation interventions	1R01CA155369-01A1	R01	NCI	$775,677
Crowley, Rebecca S. et al.	Implementation of automated guideline adherence feedback in Malawi	1R03TW009217-01A1	R03	FIC	$74,775
Dolcini, M. Margaret	Influences on translation of an evidence-based HIV/STI intervention into practice	1R01MH085502-01	R01	NIMH	$1,452,624
Dorsey, Shannon	Improving practice in community-based settings: a randomized trial of supervision	1R01MH095749-01	R01	NIMH	$590,142
Dowdy, David Wesley	A user-friendly epidemic-economic model of diagnostic tests for tuberculosis	1R21AI101152-01	R21	NIAID	$243,000
Dunn, Andrea L.	Study of the naturalistic dissemination process of an evidence-based program	1R01HL086448-01	R01	NHLBI	$1,386,185
Epstein, Jeff N.	Disseminating a model intervention to promote improved ADHD care in the community	1R21MH082714-01	R21	NIMH	$371,250
Feldstein, Adrianne C.	Forging implementation of cancer screening reminder systems (FICSRS)	1R21CA124395-01A1	R21	NCI	$357,432
Feldstein, Adrianne C.	Focusing implementation to bring effective reminders: FIBER	1R01CA132709-01	R01	NCI	$2,271,788
Foley, Kristie L.	Implementation and dissemination of tobacco cessation strategies in free clinics	1R21DA024631-01	R21	NIDA	$370,064
Friedland, Gerald H. et al.	Implementing point-of-care CD4 analysis to decentralize HIV care in rural Africa	1R21AI102756-01	R21	NIAID	$208,978
Glisson, Charles A.	Testing an organizational implementation strategy in children's mental health	1R01MH084855-01A1	R01	NIMH	$2,448,880
Hahn, Ellen J.	An intervention for promoting smoke-free policy in rural Kentucky	1R01HL086450-01	R01	NHLBI	$3,393,577
Hannon, Margaret A.	Workplace health promotion	1R21CA136435-01A1	R21	NCI	$303,019
Hannon, Margaret A.	Increasing implementation of evidence-based interventions at low-wage worksites	1R01CA160217-01A1	R01	NCI	$500,557
Hansen, William B.	The impact of adaptation on successful implementation	5R01DA024639-02	R01	NIDA	$1,176,751
Hawkins, Robert P.	Implementing CHESS ehealth breast cancer support in population-based care	1R01CA149005-01A1	R01	NCI	$1,000,078
Hawley, Kristin M.	Increasing the capacity of providers to monitor fidelity to child and family CBT	1R21MH090460-01A1	R21	NIMH	$402,298
Holt, Cheryl L.	Implementation of evidence-based cancer early detection in black churches	1R01CA147313-01A1	R01	NCI	$967,626
Ibrahim, Jennifer K.	Translating science into policy: a survey of state tobacco control plans	1R03CA128644-01A1	R03	NCI	$150,00
Kataoka, Sheryl H.	Implementation strategy for delivering a school-based mental health program	1R21MH082712-01A1	R21	NIMH	$454,805
Kendall, Philip C.	Disseminating evidence-based practice to the schools: CBT for child anxiety	1R01MH086438-01A2	R01	NIMH	$996,912
Krein, Sarah et al.	Implementing evidence to prevent urinary infection and enhance patient safety	1R01NR010700-01	R01	NINR	$1,715,510
Kruk, Margaret E.	Improving maternal and newborn health using the HIV/AIDS program platform in Tanzania	1R01AI093182-01A1	R01	NIAID	$1,499,244
Larkey, Linda K. et al.	Navigation from community to clinic to promote population CRC screening in underserved population	1R01CA162393-01A1	R01	NCI	$639,914
Leischow, Scott J.	Knowledge integration in quitlines: networks that improve cessation	1R01CA128638-01A1	R01	NCI	$3,118,026
Lounsbury, David William	Dynamics modeling as a tool for disseminating the PHS tobacco treatment guideline	1R03DA022278-01A1	R03	NIDA	$177,913
Magura, Stephen	Critical review of evidence-based program repositories for behavioral health treatment	1R21DA032151-01	R21	NIDA	$409,750
Mold, James W.	Implementation of asthma guidelines in primary care; comparison of 4 approaches	1R01HL091827-01A2	R01	NHLBI	$1,680,075
Molfenter, Todd David	To test a payer/treatment agency intervention to increase use of buprenorphine	1R01DA030431-01A1	R01	NIDA	$1,199,218
Mullen, Patricia Dolan et al.	Increasing reach and implementation of evidence-based programs for cancer control	1R01CA163526-01	R01	NCI	$653,871
Nahm, Eun-Shim	Dissemination of a theory-based bone health program in online communities	1R01NR011296-01	R01	NINR	$1,836,146
Novins, Douglas K.	Evidence-based practices and substance abuse treatment for Native Americans	1R01DA022239-01A1	R01	NIDA	$1,397,906
Nutting, Paul A.	Practice redesign to improve depression care-PRIDE care	1R01MH069806-01A2	R01	NIMH	$1,169,532
Ouslander, Joseph G. et al.	Implementing interventions to reduce hospitalizations of nursing home residents	1R01NR012936-01A1	R01	NINR	$880,217
Pankratz, Melinda M.	Comparing multiple methods of measuring fidelity of curriculum implementation	1R21DA025588-01	R21	NIDA	$427,262
Picone, Gabriel	Social interactions and malaria preventive behaviors in sub-Saharan Africa	1R03TW009108-01	R03	FIC	$147,000
Powell, Adam A.	Measurement of inappropriate screening tests (MIST)	1R03CA166719-01A1	R03	NCI	$63,000
Prudhomme O'Meara, Wendy	Sustainable financial incentives to improve prescription practices for malaria	1R21AI095979-01A1	R21	NIAID	$177,020
Psoter, Walter J.	Increasing oral cancer screening by dentists: qualitative research on practitioner	1R21DE019766-01A1	R21	NIDCR	$687,073
Reid, Manney Carrington	Implementing a cognitive/exercise therapy for back pain in the community setting	1R03NR010093-01	R03	NINR	$166,404
Rush, William Adams	An innovative approach to disseminate dental research	1R01DE022332-01	R01	NIDCR	$935,330
Sahler, Olle Jane Z.	Online problem-solving skills training for mothers of childhood cancer patients	1R01CA159013-01A1	R01	NCI	$718,361
Shelley, Donna R. et al.	Implementing tobacco use treatment guidelines in dental public health clinics	1R01CA162035-01A1	R01	NCI	$700,817
Simon, Melissa Andrea	Adapting patient navigation to promote cancer screening in Chicago's Chinatown	1R01CA163830-01	R01	NCI	$602,190
Smith, Selina A. et al.	Efficacy-to-effectiveness transition of an educational program to increase colorectal cancer screening	1R01CA166785-01	R01	NCI	$449,429
Solberg, Leif I.	Evaluation of a natural experiment to improve statewide depression care in MN	1R01MH080692-01	R01	NIMH	$3,219,406
Spallek, Heiko	Implementing research findings and evidence-based interventions into real-world	1R21DE021494-01	R21	NIDCR	$416,625
Squires, Daniel	Training drug treatment providers to adopt evidence-based practices	1R21DA021150-01A1	R21	NIDA	$411,255
Sutfin, Erin L.	Implementing evidence-based tobacco cessation strategies in campus health clinics	1R21CA161664-01	R21	NCI	$364,553
Tu, Shin-Ping	Cancer control dissemination to Asian Americans	1R01CA124397-01A1	R01	NCI	$987,766
Tu, Shin-Ping	Dissemination through community health centers serving diverse populations	1R21CA136460-01A1	R21	NCI	$317,884
Van Rie, Annelies T. A.	Optimizing the impact of XPERT MTB/RIF on treatment outcomes of drug-resistant TB	1R01AI099026-01	R01	NIAID	$593,925
Vavilala, Monica Shanta	Implementation science to increase use of evidence-based pediatric brain injury	1R01NS072308-01	R01	NINDS	$1,586,221
Weiner, Bryan Jeffrey	Implementing systemic interventions to close the discovery-delivery gap	1R01CA124402-01A1	R01	NCI	$2,815,728
Weiner, Bryan Jeffrey	Increasing colorectal cancer screening rates in community health centers	1R21CA161657-01	R21	NCI	$334,068
Wells, Kenneth B.	Community partners in care	1R01MH078853-01A1	R01	NIMH	$5,996,233
Whitten, Pamela	Implementation of a telepsychiatry program in rural oncology clinics	1R21MH080699-01A2	R21	NIMH	$418,337
Wilfley, Denise Ella et al.	Implementation of evidence-based treatments for on-campus eating disorders	1R01MH095748-01	R01	NIMH	$771,666
Windsor, Richard Anthony	The West Virginia smoking cessation or reduction in pregnancy treatment trial	1R01CA124429-01A1	R01	NCI	$1,740,688
Wyatt, Gail E.	Implementing EBAN II: an evidence-based intervention for serodiscordant couples	1R01MH093230-01A1	R01	NIMH	$687,933

*This funding amount represents awards from 2005–2012. Out-year funding for projects recently awarded is not included.

NCI: National Cancer Institute; NIMH: National Institute of Mental Health; NIDA: National Institute on Drug Abuse; NHLBI: National Heart, Lung, and Blood Institute; NIAID: National Institute of Allergy and Infectious Diseases; NINR: National Institute of Nursing Research; NIDCR: National Institute of Dental and Craniofacial Research; NINDS: National Institute of Neurological Disorders and Stroke; FIC: Fogarty International Center.
